# Tactile thermal oral stimulation increases the cortical representation of swallowing

**DOI:** 10.1186/1471-2202-10-71

**Published:** 2009-06-30

**Authors:** Inga K Teismann, Olaf Steinsträter, Tobias Warnecke, Sonja Suntrup, Erich B Ringelstein, Christo Pantev, Rainer Dziewas

**Affiliations:** 1 Department of Neurology, University of Muenster, Albert-Schweitzer-Str.33, 48149 Muenster, Germany; 2 Institute for Biomagnetism and Biosignalanalysis, University of Muenster, Malmedyweg 15, 48149 Muenster, Germany

## Abstract

**Background:**

Dysphagia is a leading complication in stroke patients causing aspiration pneumonia, malnutrition and increased mortality. Current strategies of swallowing therapy involve on the one hand modification of eating behaviour or swallowing technique and on the other hand facilitation of swallowing with the use of pharyngeal sensory stimulation. Thermal tactile oral stimulation (TTOS) is an established method to treat patients with neurogenic dysphagia especially if caused by sensory deficits. Little is known about the possible mechanisms by which this interventional therapy may work. We employed whole-head MEG to study changes in cortical activation during self-paced volitional swallowing in fifteen healthy subjects with and without TTOS. Data were analyzed by means of synthetic aperture magnetometry (SAM) and the group analysis of individual SAM data was performed using a permutation test.

**Results:**

Compared to the normal swallowing task a significantly increased bilateral cortical activation was seen after oropharyngeal stimulation. Analysis of the chronological changes during swallowing suggests facilitation of both the oral and the pharyngeal phase of deglutition.

**Conclusion:**

In the present study functional cortical changes elicited by oral sensory stimulation could be demonstrated. We suggest that these results reflect short-term cortical plasticity of sensory swallowing areas. These findings facilitate our understanding of the role of cortical reorganization in dysphagia treatment and recovery.

## Background

Human swallowing is a complex neuromuscular procedure modulated by sensory feedback [1,2]. Impairments of sensation have been implicated in aspiration after stroke [3-7] and are known to result in short-term dysphagia even in healthy subjects when induced by oropharyngeal anaesthesia [8,9]. While many patients experience recovery of swallowing within the first few weeks after stroke, 40% of dysphagic stroke patients develop aspiration pneumonia which in turn increases the use of artificial feeding, length of hospital stay, and mortality [10]. Despite the high incidence of aspiration pneumonia after stroke, treatment options for accelerating the recovery of swallowing by improving physiology and reducing aspiration remain limited. Current strategies of swallowing therapy involve on the one hand modification of either eating behaviour or swallowing technique and on the other hand facilitation of swallowing with the use of TTOS.

The anterior faucial pillars (AFP) are bilaterally located on the oral side of the velum and form part of the soft palate. They are innervated by the maxillary branch of the trigeminal nerve and the glossopharyngeal nerve. About 80 years ago sensory stimulation was first advocated as a method for facilitating swallowing [11]. Since then stimulation of the AFP and other parts of the oropharynx became a common treatment for dysphagia [12-15]. Clinical studies showed that tactile stimulation of the AFP increases swallowing speed and facilitates deglutition for several minutes. Different groups using electrical stimulation even found a better outcome in stroke patients showing reduced aspiration [16] and a decrease of gastrostomies [17] while others found no changes in laryngeal closure, pharyngeal transit time or aspiration severity [18]. Until now, the underlying basic physiological consequences induced by oropharyngeal stimulation are still unknown [19]. First results in this field of research revealed an increased cortical excitability evoked by pharyngeal stimulation [20,21].

Magnetoencephalography (MEG) can monitor cortical activity with a high temporal and spatial resolution [22]. Motor tasks have been shown to result in event-related desynchronisations (ERD) of the cortical beta rhythm in cortical motor areas [23,24]. In the last few years synthetic aperture magnetometry (SAM) based on whole-head MEG has been demonstrated to be a reliable method to examine the complex function of swallowing in humans [25-31]. While the artifacts caused by oropharyngeal muscle activation during the act of swallowing make it difficult to study activation in subcortical and bulbar structures, the cortical areas especially the sensorimotor areas can be examined in detail.

In the present study we employed whole-head MEG and SAM analyses to study cortical activity during self-paced volitional swallowing with and without preceding TTOS. This simple stimulation paradigm was chosen due to its non invasivness and its easy bedside application. We hypothesized an increased swallowing related activation of the somatosensory cortex after oropharyngeal stimulation compared to the baseline condition without prior stimulation.

## Results

### Behavioral data

All participants tolerated the study without any difficulty. No coughing and, in particular, no signs of aspiration occurred during stimulation or measurements. The two conditions, after and without TTOS, did not differ in swallowing behaviour. The amount of water swallowed during the two compared measurements was identical for each subject. Number of swallows (normal swallowing: 39 – 141 swallows in 15 min, mean 73.5; oral stimulation: 41 – 139, mean 73.7; p = 0.774) as well as duration per swallow (1.13 – 2.88 s, mean 2.06 s, oral stimulation: 1.37 – 2.68; mean: 2.15; p = 0.7945) did not differ between the two tasks. RMS of EMG amplitude across the swallow interval (M0 – M2) showed no difference in EMG power by comparison swallowing after and without oropharyngeal stimulation (p = 0.8347).

### Time-frequency plots

**Figure 1:**
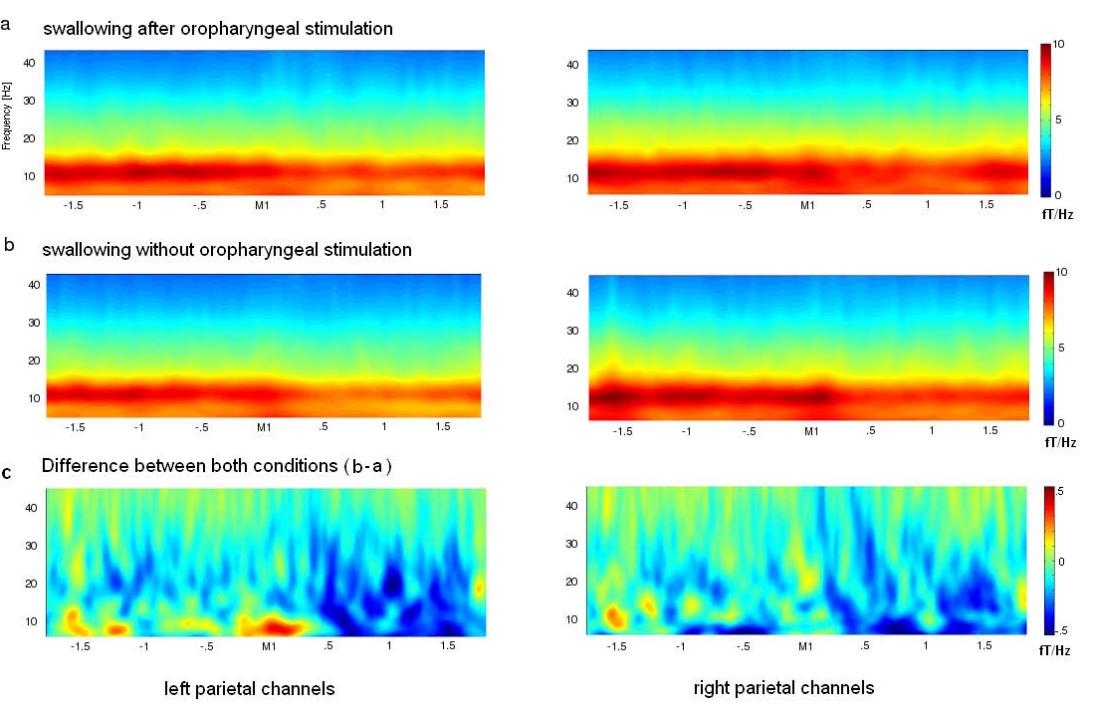
**Wavelet analysis of the parietal areas**. X-axis represents time in seconds related to M1. Y-axis represents frequencies in Hertz. a + b) In both conditions distinct activation in the higher alpha and lower beta frequency band can be seen with a reduction of activation at about M1 and a re-increase after about 400 – 600 ms in both hemispheres. c) The difference plot of both measurements (without oropharyngeal stimulation minus with oropharyngeal stimulation) reveals variations mainly during deglutition (after M1). Similar activation is found in both hemispheres before swallowing onset. Colors represent the level of frequency power (fT/Hz), with lower numbers (blue) indicating a decrease in power (ERD) and higher numbers (red) an increase in power (ERS). In the difference plot blue corresponds to stronger activation in the measurement after stimulation, while red demonstrates stronger activation in the condition without stimulation.

Wavelet group analysis of MEG sensor recordings revealed distinct activation in the higher alpha and lower beta frequency band in the parietal sensors with a reduction of activation at about M1 and a re-increase after about 400 – 600 ms. This effect was observable in both hemispheres and conditions (see figure 1a, b). A difference plot of both conditions demonstrates stronger desynchronization in the stimulation condition compared to the reference measurement (see figure 1c). According to these results MEG data were then filtered in the alpha and beta band.

### SAM Analysis

**Figure 2:**
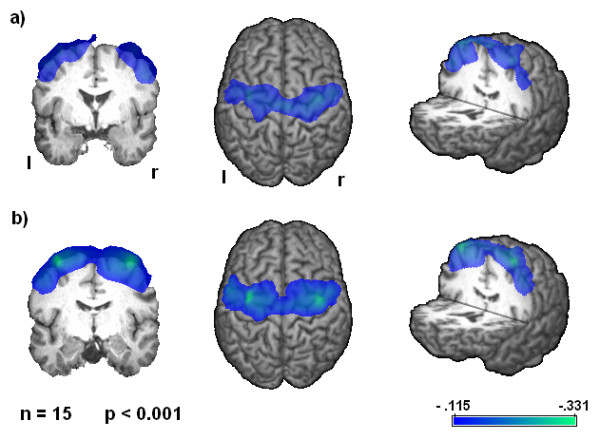
**Event related desynchronization**. Significant activation in group analysis is shown (p < 0.001). Changes in the beta-frequency-band during swallowing execution compared to the resting stage. a) Significant cortical activation after volitional swallowing without oropharyngeal stimulation. b) Cortical swallowing activation after oropharyngeal stimulation is broader in both hemispheres. The color bar represents the t-value.

Group analysis of SAM results revealed significant event related desynchronizations (ERD) in the beta frequency band located in the primary sensorimotor cortex (BAs 4, 3, 1, and 2) in both conditions (p < 0.05) (see figure 2). The peak of the ERD was located bilaterally in the same area around the central gyrus in both conditions. In the alpha frequency band and other cortical areas no significant activation was observed in either of the two conditions. Comparison of both conditions revealed a significantly stronger activation after TTOS compared to the normal swallowing task (p < 0.05). The maximum pseudo-t value increased in the TTOS condition (34.1% in the right hemisphere, 13.6% in the left hemisphere).

**Figure 3:**
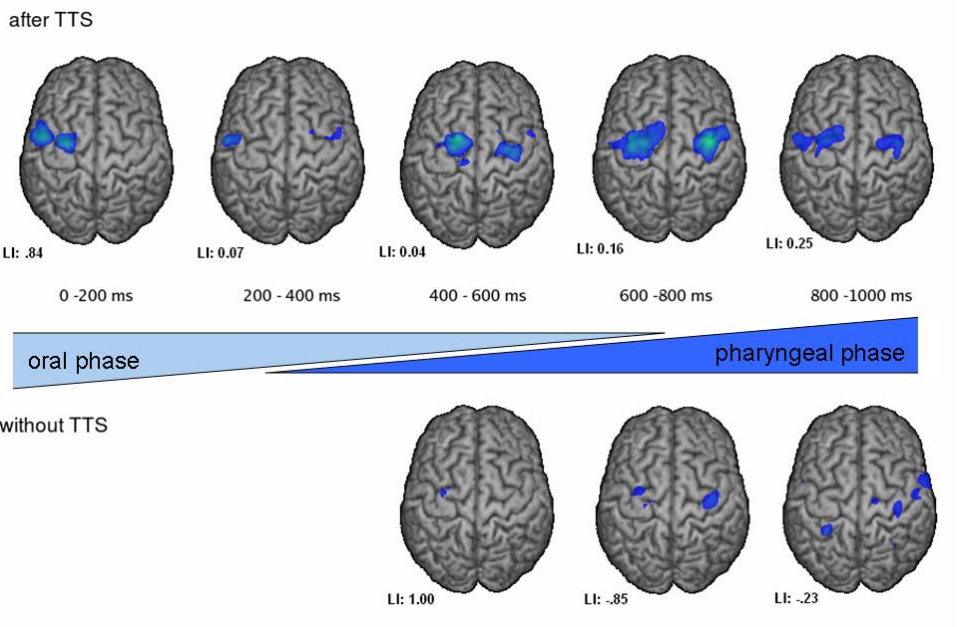
**Event related desynchronizations in the beta frequency band during the five successive 200 ms time intervals of the swallowing execution phase is shown for both groups**. Significant activation in the group analysis is shown (p < 0.05). Estimation of the according swallowing phase is shown. The color bar represents the t-value. The lateralization index for each individual time interval and for both conditions is indicated.

To analyze the chronological changes during swallowing separate calculation of SAM images for each 200 ms interval was calculated. Here the early intervals represent the oral phase of deglutition while the later intervals are part of the pharyngeal swallowing phase. A clear distinction between the two phases based on the submental EMG recordings is not possible. This revealed ERD of rhythmic brain activity within sensorimotor cortex in each individual subject and interval. Group analysis of the normal swallowing paradigm showed no significant activation during the first 400 ms. Only small left sided activation appeared in the third time interval. Between 600 ms and 1 s right hemispheric lateralization of activation could be observed. In contrast, after TTOS significant left lateralized activation was seen in all 5 time intervals. Right hemispheric activation increases over time with a slight decrease in the last time frame (see figure 3).

## Discussion

The present study revealed an increment of cortical swallowing activation after TTOS in healthy subjects. By this physiological changes on the cortical level induced by this widely used tool of dysphagia rehabilitation are shown.

### Behavioural changes after oropharyngeal stimulation

Different behavioural studies examined the effect of oropharyngeal stimulation before. Either thermal or taste stimuli are supposed to heighten the sensitivity for swallowing in the oral cavity thereby leading to a more rapid triggering of the swallowing reflex [19]. Today this technique is often used in the treatment of patients with neurogenic dysphagia to facilitate a delayed or absent swallowing response. There is little data reporting the effectiveness of this therapy.

Cold stimulation of the AFP before swallowing hastened the onset of the pharyngeal swallowing phase and reduced the swallowing latency [32,33]. Lazzara and co-workers could show that TTOS on 25 patients with different neurologic diseases resulted in an improved triggering of the swallowing reflex in 23 of these patients [33]. Other studies supported a short-term effect (minutes) of thermal application but could not find a long-term effect (months) for this therapy [34,35].

Also taste stimuli have been shown effects on swallowing. They reduced the delay in swallowing initiation, hastened triggering of pharyngeal swallowing in patients with neurogenic dysphagia and even led to a reduced frequency of radiographically observed aspiration. This suggests that afferents from the oral-pharyngeal chemoreceptors can facilitate deglutition [36].

### Cortical changes after oropharyngeal stimulation

Additionally to the observed and well known behavioural changes following oropharyngeal stimulation few studies focussed on its effects regarding the cortical level. The first study focusing on this topic in 1997 demonstrated a facilitation of the cortical pathways by cranial nerve stimulation [37]. Apart from that, electrical pharyngeal stimulation showed an increase of cortical excitability in different TMS studies [20,38]. To our knowledge the cortical reaction to TTOS has not been examined yet. In the present study a significant increase of cortical swallowing activation was observed after TTOS compared to a swallowing paradigm without stimulation. These findings demonstrate cortical changes following simple oral stimulation. Analysis of the chronological changes during the swallowing execution might provide further insights into the underlying physiological mechanisms. In healthy subjects a time-dependent shift from the left to the right hemisphere was found in an MEG swallowing paradigm [29]. Though from the submental EMG data no clear cut between oral and pharyngeal phase can be defined, it is likely that the beginning of submental muscle activation represents at least part of the oral phase, while about 500 ms later and in the end of the recorded submental muscle activation the pharyngeal phase is taking place.

Although in the present experiment SAM analysis of the first two 200 ms intervals did not reveal significant activation in either hemisphere, an increase of right hemispheric activation was seen in the following time intervals. Therefore the results of the normal swallowing condition found in the present study are mainly concordant with the previous investigation. In contrast to this, TTOS revealed increased bihemispheric activation with predominant activation of the left somatosensory cortical areas during the whole swallowing interval. This finding underlines the hypothesis of hemispheric specialization in swallowing processing. In lesion studies left hemispheric infarction was associated with oral stage dysfunction, while dysfunction of the pharyngeal stage was related to right hemispheric lesions [39,40]. Based on their findings, Daniels and co-workers suggested a left hemisphere control for volitional aspects of swallowing and a right hemisphere control for reflexive swallowing behaviour. This is also supported by the MEG study of our group mentioned above [29]. Finally, patients with a chronic pharyngeal stage dysfunction revealed stronger right hemispheric activation, both in size and time, indicating cortical compensation of their pharyngeal dysphagia [41].

Along this reasoning, TTOS, according to the present findings, may lead to a facilitation of both the oral and the pharyngeal phase of deglutition. On the other hand it remains unclear whether the observed effects are related to functional cortical reorganization or are more unspecific reactions to differences in attention due to the afferent input. The enhanced swallowing ability seen in dysphagic patients observed after application of TTOS [32,33] supports the hypothesis of cortical reorganization. However, based on the present study we cannot distinguish whether the observed effects are caused by the stimulation of the AFP or only by the swallowing of a chilled bolus.

Our results and their interpretation are also supported by behavioural studies employing TTOS showing both changes of oral phase tasks, like a heightened sensitivity of the oral cavity [19] and a reduced delay in swallowing initiation, and modification of the pharyngeal phase, like an improved triggering of the swallowing reflex [32,33]. Though long term changes in swallowing behavior after TTOS could not be shown yet, our findings may point to therapeutical approaches in swallowing rehabilitation. Further studies have to show if stimulation intensity, frequency or treatment duration lead to different results in swallowing behavior and in the consecutive cortical activations. Additionally cortical and behavioral changes of TTOS have to be examined in dysphagic patients.

## Conclusion

In the present study we could demonstrate an increase of cortical activation after thermal tactile oral stimulation. This is to our knowledge the first study showing cortical changes elicited by this simple swallowing therapy technique. Our results provide an insight into the physiological mechanisms by which TTOS might lead to the previously observed facilitation of swallowing. Further examinations employing TTOS in dysphagic patients have to show that increased cortical activation is paralleled by an improved swallowing performance.

## Methods

### Subjects

Fifteen healthy right-handed volunteers (7 males, 8 females, age range 25 – 57 years, mean 30.4 years) served as subjects. The local regional ethics committee approved the protocol of the study. Informed consent was obtained from each subject after the nature of the study was explained in accordance to the principles of the Declaration of Helsinki (2008).

### Tactile Thermal Oral Stimulation (TTOS)

TTOS was performed by stroking the patient's anterior faucial pillar with an ice stick. The surface temperature of the stick was between -1° and 3°C. Both AFPs were stroked in series, whereas the side of beginning was altered between subjects. Stroke direction was from top (medial) to bottom (lateral). We took care that the tongue was not at all touched by the ice stick. After stroking both sides three times subjects were instructed to swallow to eliminate the melt water. This was done 5 times within 2 minutes. This procedure was performed directly before the corresponding MEG measurement. Due to the startup procedure of the MEG system the overall time between stimulation and the beginning of the measurements was between 2 and 3 minutes.

To eliminate a bias due to the forced swallows directly before measurement subjects were instructed to swallow 5 times about 3 minutes before the beginning of the MEG recording in the condition without TTOS.

### Intraoral infusion

To facilitate volitional swallowing during MEG recording water was infused into the oral cavity via a flexible plastic tube 4.7 mm in diameter attached to a fluid reservoir. The reservoir bag was positioned about 1 m above the mouth of each subject when seated. The tip of the tube was placed in the corner of the mouth between the buccal part of the teeth and the cheek. The tube was gently fixed to the skin with tape. The side chosen for tube placement was alternated between subjects but consistent in each subject. The infusion flow was individually adjusted to the subject's request and ranged between 8 and 12 ml/min. The aim was to establish a swallowing frequency of four to six times per minute. This resulted in a swallowing volume of about two to three ml. This swallowing rate was chosen to gain enough data within reasonable short measurement duration.

### MEG recording

In each MEG measurement of 15 min duration subjects swallowed self-paced without external cue while swallowing acts were recorded and identified by electromyographic recording. The MEG recording was done with and without oral stimulation in all 15 subjects investigated. In 8 subjects the normal swallowing task was done first, the other 7 started with oral stimulation. In each subject, both measurements were 14 days apart.

MEG data were collected using a whole head 275-channel SQUID sensor array (Omega 275, CTF Systems Inc.). Magnetic fields were recorded with a sample frequency of 600 Hz. The data were filtered during acquisition using a 150 Hz low-pass filter. Recordings were performed while subjects were seated in a comfortably upright position and watching a self selected silent movie.

By this the level of attention was kept stable and avoided falling asleep. Vision should be focused on the video screen during measurement to reduce eye movement artifacts. No confound by the movie was expected due to its continuous presentation during deglutition and the resting stages.

### EMG recording

Submental recording of muscle activation is a simple and reliable noninvasive screening method for evaluating swallowing with low levels of discomfort [42]. Another advantage compared to needle EMG is the broader muscle spectrum that can be recorded. Disadvantages are higher inter- and intraindividual variability and a higher artifact rate [43]. Surface EMG was measured with two pairs of bipolar skin electrodes (Ag-AgCl) placed on the submental muscle groups [42,44]. The electrodes were connected to a bipolar amplifier (DSQ 2017E EOG/EMG system, CTF Systems Inc., Canada), and the nominal gain was set at 1. EMG data was high pass filtered with 0.1 Hz before markers were manually set.

### Anatomical MRI

MRI data were acquired on a 3.0 T Scanner (Gyroscan Intera, Philips Medical Systems, Best, The Netherlands) with a standard head coil. T1-weighted sagittal anatomical images with in-plane resolution of 512 × 512 (0.6 × 0.6 mm resolution) and 320 slices (0.5 mm thickness) were recorded using spoiled gradient echo imaging.

### Data analysis

**Figure 4:**
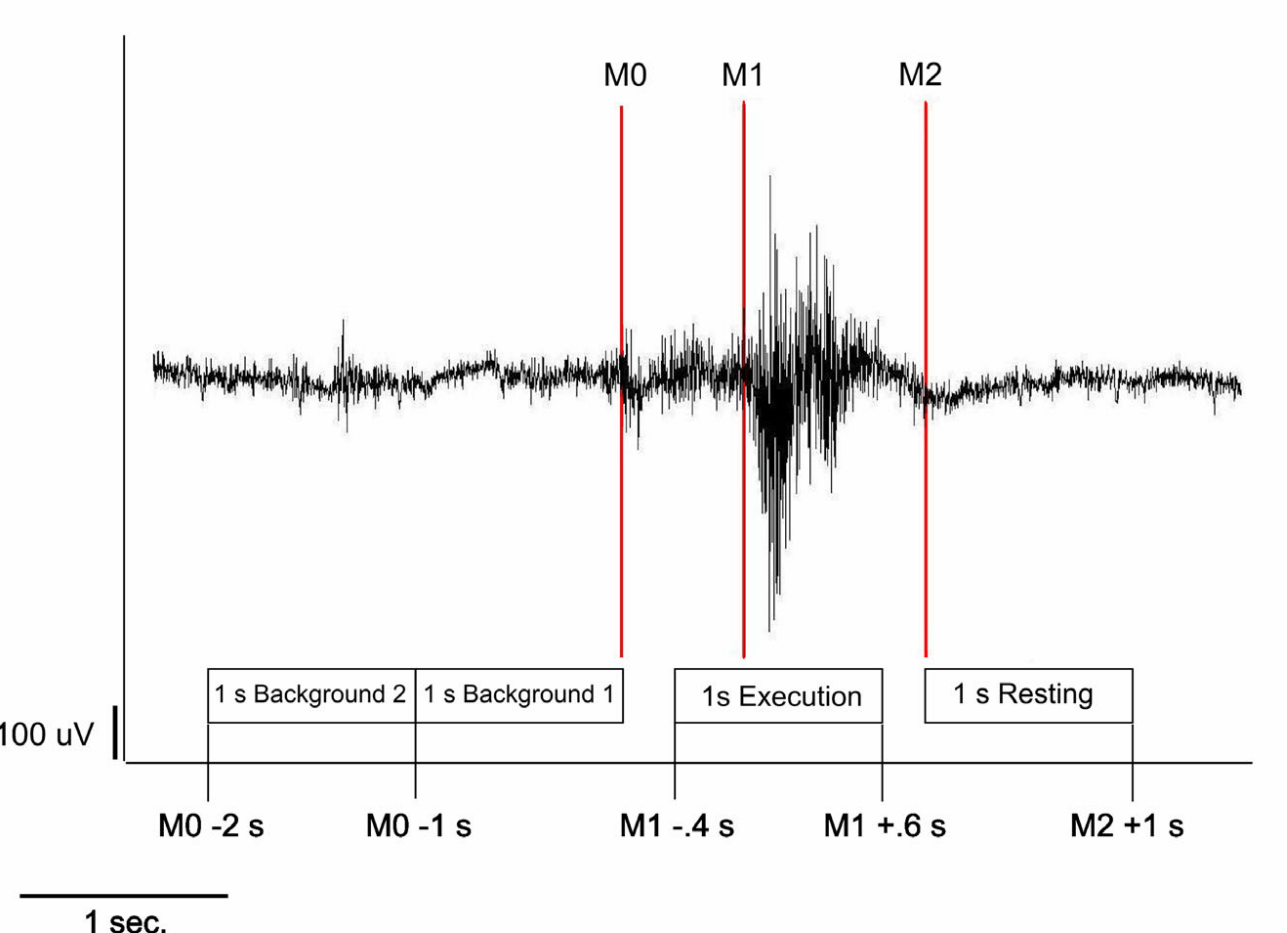
**EMG recording and resulting time phases**. Definition of active, resting and background stages of swallowing-related muscle activity. The EMG recording of one swallowing act is shown (surface electrodes, recording from the submental muscles). To distinguish the swallowing execution phase, each individual's EMG signal was used to mark the swallowing related muscle activation. After an initial increase of more than 50% of EMG activity, the beginning of main muscle activation (M1; 100% increase of activation) and the end of swallowing specific muscle activation (M2; 50% decrease of activation) were marked. To estimate the maximal null distribution a third marker (M_0_) at the beginning of preparation activity was set and two background phases were defined (see methods section).

Each individual's EMG signal was used to mark the beginning of main muscle activation (M_1_) and the end of the task-specific muscle activity (M_2_) for every single swallow (see figure 4). The beginning of the main muscle activation was defined as an enduring > 100% increase in amplitude or frequency of the EMG signal after an initial increase of more than 50% of EMG activity defining the onset of swallowing preparation. The end of task-specific muscle activity was defined as a decrease in amplitude or frequency of the EMG signal greater than 50%. To estimate the maximal null distribution (see below), a third marker was set to distinguish background activity from the onset of swallowing preparation (M_0_). The examiner who set the markers to the datasets was blinded to the two tasks. For further analysis time intervals were defined as following:

(1) Movement stage: -0.4 to 0.6 s in reference to M_1_

(2) Resting stage: 0 to 1 s in reference to M_2_

(3) Background stage 1: -2 to -1 s in reference to M_0_

(4) Background stage 2: -1 to 0 s in reference to M_0_

About five percent of the trials were rejected due to overlap between (1) and (2) or between (4) and (2) of the subsequent swallow. The time intervals of (3) and (4) were used to estimate the maximum null distribution. To define the active frequency bands and to examine the temporal sequencing of activation time-frequency plots were calculated using wavelet analysis. These calculations were done using EMEGS (ElectroMagnetic-EncephaloGraphy Software; ), a tool for analyzing neuroscientific data developed in MATLAB [45]. The 275 channels of the MEG system were fragmented into 10 channel groups, frontal, central, parietal, temporal and occipital channels in each hemisphere. Data from each individual subject was averaged across trials (-2 to 2 s in reference to M_1_) and time-frequency analysis was performed (0 – 150 Hz). The time-frequency plots of the parietal channels were determined for both hemispheres and averaged across all subjects in each group. Afterwards the two time intervals "execution" (1) and "predeglution" (5) were defined for further calculations. These intervals were chosen because they are both in reference to M1. Therefore a direct comparison is possible without further calculations. Comparisons between the two time intervals, the two hemispheres, and the two groups were performed using two-way ANOVA followed by post-hoc t-tests.

According to the changes of the time-frequency analysis MEG data were than filtered within two frequency bands: alpha (8–13 Hz) and beta (13–30 Hz). From the filtered MEG data, SAM was used to generate a 20 × 20 × 14 cm volumetric pseudo-t images [46] with 3 mm voxel resolution for both frequency bands. A pseudo-t value cancels the common-mode brain activity by subtracting the source power found in a defined control stage from the source power in the active stage. To account for uncorrelated sensor noise, this difference is normalized by the mapped noise power [46]. Data from the execution stages described above were used to analyze cortical activity during the different time intervals. The corresponding resting stage served as a control.

**Figure 5:**
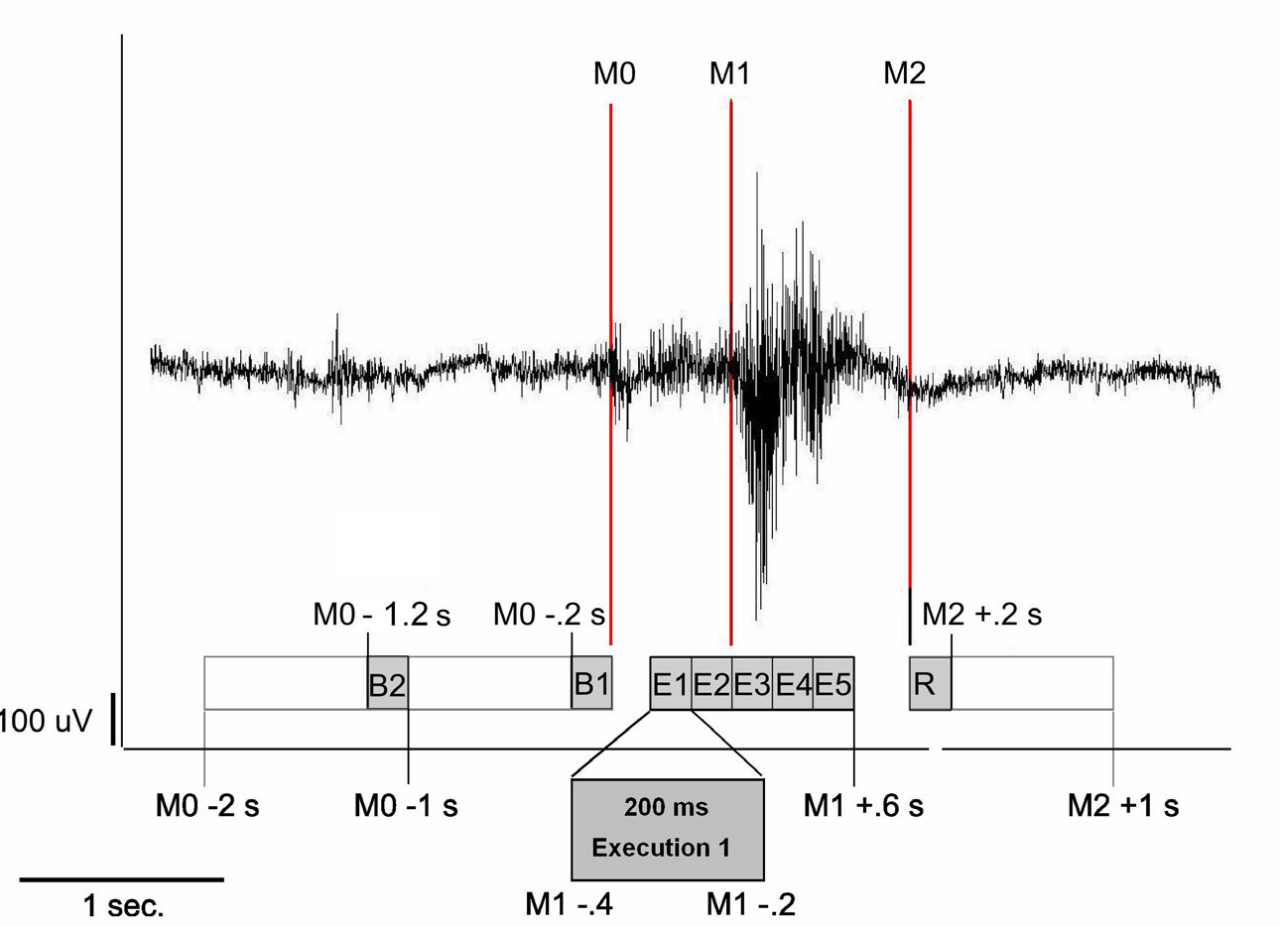
**EMG recording with division of the execution stage**. To analyze the cortical activation within the early and later stages of the execution phase, this 1 second interval is divided into 5 successive 200 ms time intervals (E1 – E5). The corresponding resting stage (R) and two background stages (B1 and B2) are also shortened to 200 ms (Methods).

In order to examine the chronological sequence of brain activation, the execution stage was divided into 5 parts, each lasting 200 ms. Time intervals including the according resting stages for the subsequent analysis were defined as follows (see figure 5):

(1) 200 ms Execution stage 1 (E1): -0.4 to -0.2 s in reference to M_1_

(2) 200 ms Execution stage 2 (E2): -0.2 to 0.0 s in reference to M_1_

(3) 200 ms Execution stage 3 (E3): 0.0 to 0.2 s in reference to M_1_

(4) 200 ms Execution stage 4 (E4): 0.2 to 0.4 s in reference to M_1_

(5) 200 ms Execution stage 5 (E5): 0.4 to 0.6 s in reference to M_1_

(6) 200 ms Resting stage (R): 0 to 0.2 s in reference to M_2_

(7) 200 ms Background active (B1): -0.2 to 0 s in reference to M_0_

(8) 200 ms Background control (B2): -0.4 to -0.2 s in reference to M_0_

Group analysis of multiple subjects' data was performed as previously published [47-50]. Briefly, the individual MRIs were first transformed into a common anatomical space using SPM2. Then the spatial normalized activation maps were obtained by applying this transformation to the individual SAM volumes. The significance of activated brain regions was investigated by the permutation test method described by Chau and co-workers (2004). The maximal null distribution was estimated by comparing the two background stages (3) and (4) [50,51]. The required similarity between the resting stage and the two background stages in both examined groups was proven before by a direct comparison of these 3 stages. For comparison of both conditions a standard permutation test for unpaired samples was performed [51].

Hemispheric lateralization concerning the five different time intervals of swallowing related activation was quantified using a lateralization index (LI), which was calculated as (L-R)/(L+R), where L and R are the cumulative pseudo-t activation in the sensorimotor cortex (BA 3, 1, 2 and 4, according to the Talairach atlas) of the left and right hemisphere, respectively. A positive LI indicates left hemispheric lateralization, while a negative LI indicates stronger right hemispheric activation. A LI of about 0 represents indeterminate dominance, 1, respectively -1 are indicating unilateral activation [26,52].

## Authors' contributions

IT performed analysis and interpretation of data and drafted the manuscript. She was funded by the Deutsche Forschungsgemeinschaft. OS has made analysis and interpretation of data and was involved in drafting the manuscript. He was funded by Deutsche Forschungsgemeinschaft. TW and SS have made contributions to conception and design and did data acquisition. EBR and CP revised the manuscript critically for important intellectual content. RD made substantial contributions to conception and design, and has given final approval of the version to be published. All authors read and approved the final version of the manuscript.
